# Brain microvascular endothelial cells possess a second cilium that arises from the daughter centriole

**DOI:** 10.3389/fmolb.2023.1250016

**Published:** 2023-11-06

**Authors:** Karthikeyan Thirugnanam, Ankan Gupta, Francisco Nunez, Shubhangi Prabhudesai, Amy Y. Pan, Surya M. Nauli, Ramani Ramchandran

**Affiliations:** ^1^ Department of Pediatrics, Division of Neonatology, Developmental Vascular Biology Program, Medical College of Wisconsin, Children’s Research Institute (CRI), Milwaukee, WI, United States; ^2^ Department of Pharmaceutical Sciences, Chapman University, Irvine, CA, United States; ^3^ Department of Pediatrics, Division of Quantitative Health Sciences, Medical College of Wisconsin, Children’s Research Institute, Milwaukee, WI, United States

**Keywords:** PKD2, brain endothelial cell, cilia, cell cycle, G0

## Abstract

Primary cilia from the brain microvascular endothelial cells (ECs) are specialized cell-surface organelles involved in mediating sensory perception, cell signaling, and vascular stability. Immunofluorescence (IF) analysis of human primary brain microvascular ECs reveals two cilia per cell. To confirm the *in vitro* observation of the two-cilia phenotype in human primary brain ECs, ECs isolated from mouse brain were cultured and stained for cilium. Indeed, brain ECs from a ciliopathic mouse (polycystic kidney disease or *Pkd2* ^*−/−*^) also possess more than one cilium. Primary cilium emerges from the mother centriole. Centriole analysis by IF suggests that in brain ECs, markers for the mother and daughter centrioles stain both cilia, suggesting that the second cilium in brain ECs arises from the daughter centriole. Further quantification of cilia size in brain ECs revealed that cilia arising from the mother centriole are bigger in size compared with cilia from the daughter centriole. Cell cycle analyses using immunoblotting and flow cytometry suggest that the ciliary proteins ARL13B and IFT88 involved in brain EC ciliogenesis are highly expressed only in the G0/G1 and S phases of the cell cycle. The IF analyses of cells arrested at different cell cycle stages indicate that the two-cilia phenotype is highly specific to the G0/G1 phase. Our findings suggest that in addition to the mother centriole, the daughter centriole also plays a role in ciliogenesis in primary cultured ECs.

## Introduction

Primary cilia are microtubule-based organelles mainly composed of a basal body, axoneme, ciliary matrix, and ciliary membrane (Ma and Zhou, 2020). Primary cilium is found on most cells in the body, and typically, one primary cilium exists per cell. In vascular endothelial cells (ECs), primary cilia extend into the lumen of blood vessels and act as sensors and transmit extracellular signals into the cell (Satir et al., 2010). Primary cilia have many important functions in cells, and their dysfunction has been linked to multiple human pathologies, collectively called ciliopathies (Fry et al., 2014). Mechanistically, during ciliogenesis, at the end of mitosis, when the cell enters the G0/G1 phase of the cell cycle, the basal body of primary cilium arises from the mother centriole. Centrioles are microtubule-based structures in eukaryotic cells that exist as a pair (mother and daughter centriole) and play an important role in all stages of the cell cycle. For example, during interphase, the mother centriole functions to assemble primary cilia. The contribution of the mother centriole during primary ciliogenesis includes the assembly of distal and subdistal appendages, docking of pre-ciliary vesicles to distal appendages, formation of the ciliary vesicle, and axoneme and ciliary membrane elongation (Kumar and Reiter, 2021). Thus, the role of the mother centriole is important in primary ciliogenesis. On the other hand, apart from centrosome duplication occurring in the S phase of the cell cycle (Conduit et al., 2015), the role of the daughter centriole is poorly studied, especially in the context of primary ciliogenesis. However, a recent study suggests the proximity of the daughter centriole is important in determining primary ciliogenesis from the mother centriole (Loukil et al., 2017).

Reports in the literature have noted more than one cilium in cells, and when and how they originate in a cell is not understood. For example, islet beta cells show multiple cilia (Polino et al., 2023) and in the vasculature, brain collateral vessels show multiple primary cilia (Zhang et al., 2019), which are presumably on ECs or pericytes. Previously, we have observed two cilia in primary brain ECs *in vitro* (Thirugnanam et al., 2022). In the present study, we characterized the origin of the second cilium in human primary brain microvascular ECs (HBMECs). Our data reveals the second cilium is developing from the daughter centriole preferentially occurring in the G0/G1 phase of the cell cycle and is not independent of the mother centriole.

## Methods

### Mice brain endothelial cell isolation and staining for primary cilia

All animal procedures were performed according to the Chapman University Animal Care and Use Committee Guidelines. One-week-old *Tie2Cre·Pkd2* ^*WT/WT*^ (with Cre activation; control wild-type group) and *Tie2Cre·Pkd2* ^*flox/flox*^ (with Cre activation; experimental *Pkd2* group) mice were injected intraperitoneally with 5 μg/μL tamoxifen every day for five consecutive days. Frontal cortices were collected. Cells were dissociated with 1× trypsin/Ethylenediaminetetraacetic acid (EDTA) solution through a 1-cm^3^ 25 G⅝ needle and plated in endothelia-selecting media Dulbecco's modified eagle medium (DMEM) containing 2% Fetal bovine serum (FBS), 0.75 μg/L interferon-γ, 1.0 g/L insulin, 0.67 mg/L sodium selenite, 0.55 g/L transferrin, 0.2 g/L ethanolamine, 36 ng/mL hydrocortisone, 0.10 μmol/L 2,3,5-triido-l-thyronine, 100 U penicillin-G (base) combined with 0.30 mg/mL additional glutamine, 100 μg streptomycin sulfate, and 0.1 mmol/L citrate to maintain penicillin potency. All cell-culture supplements were obtained from Sigma-Aldrich(St Louis, Mo). After 2–3 weeks of growth, endothelial cells were further sorted by incubating the cells with 10 mg/mL of the endothelial marker intracellular adhesion molecule-2 (ICAM-2; Santa Cruz Biotechnology, Santa Cruz, Calif). Fluorescein isothiocyanate (FITC)-conjugated ICAM-2 antibody was applied for 1 h at room temperature at a dilution of 1:100 in Phosphate buffered saline (PBS) containing 1% fetal bovine serum to prevent any non-specific binding of the antibody. After the cells were washed three times to avoid non-specific binding, they were analyzed with FACScan (Becton Dickinson, Franklin Lakes, NJ) at a wavelength of 525 nm (FITC, FL-1) (Supplementary Figure S1). Following cell sorting and as needed to avoid bacteria contamination, 100 U penicillin-G (base) was added to the DMEM with 0.30 mg/mL additional glutamine, 100 μg streptomycin sulfate, and 0.1 mmol/L citrate to maintain penicillin potency. Cells were grown for an additional 4–7 days. For staining cilia structures, acetylated-α-tubulin (1:10,000, Sigma) and the secondary antibodies were also diluted in 10% FBS to decrease the background fluorescence; FITC fluorescence secondary antibody (1:1000; Pierce, Inc.) was used. Cells were then washed three times for 5 min each with cacodylate buffer and mounted with 4',6-diamidino-2-phenylindole (DAPI) (Vector laboratories). Confocal microscopic images were obtained using an inverted Nikon Eclipse Ti confocal microscope.

### Primary brain ECs cell culture conditions

Human brain microvascular ECs (HBMECs) were purchased from Cell Systems Corporation (Cat # ACBRI 376) and maintained at 37°C in 5% CO_2_ in endothelial cell complete media (Promocell, Cat #C22010). All experiments were performed with cells grown between passage 4–6. These cells were extensively characterized for EC markers in our previous publication (Thirugnanam et al., 2022). Cells were grown and maintained at 50% confluence state. IF experiments were performed at 60%–70% confluence to distinguish and avoid the overlapping of cells and cilia.

### Western blot

Proteins were isolated from HBMECs using Radioimmunoprecipitation Assay buffer (RIPA) buffer (Sigma Cat#R2078) with a complete mini EDTA-free protease inhibitor cocktail (Roche Cat#11836170001) and PhosSTOP phosphatase inhibitor (Roche Cat#4906845001). After isolation, the total protein was quantified. Cell lysates were used for probing the following proteins: ARL13B (Proteintech, Cat#17711-I-AP), IFT88 (Thermofisher, Cat#PA5-18467), Cyclin D1 (Thermofisher, Cat#MA5-16356), Cyclin A (Biolegend, Cat#644004), Cyclin B1 (Biolegend, Cat#647906), Cyclin E1 (Cell signaling, Cat#20808), CDK1 (Biolegend, Cat#626901), CEP164 (Proteintech, Cat#22227-1-AP), CENTRIN2 (Biolegend, Cat#698602), and β-actin (Cell signaling Cat#4970). Anti-rabbit Horseradish peroxidase (HRP) (Cell signaling, Cat#7074), anti-goat HRP (Jackson Immunoresearch, Cat#205-052-176), and anti-mouse HRP (Cell signaling, Cat#7076) were secondary antibodies used for chemiluminescence detection. Quantification was done using ImageJ software and plotted against the housekeeping control protein (β-actin) using GraphPad software as described previously (Thirugnanam et al., 2022).

### Primary cilia immunostaining

HBMECs were grown to confluence in six-well plates on coverslips. All the IF experiments were performed by seeding cells on the same day with similar seeding density and synchronized. Cells were washed with 1X PBS (Gibco, Cat#10010023) thrice and fixed with 4% PFA(Electron Microscopy Sciences, Cat#15710) for 15 mins. Fixed cells were washed again with 1X PBS before permeabilization with 0.1% Triton X-100 (BioRad, Cat#1610407). This was followed by blocking in 4% BSA in PBS and overnight incubation with primary antibodies of ARL13B (1:500), IFT88 (1:100), CEP164 (1:100), CENTRIN2 (1:200), NINEIN (Novus biologicals, Cat#NBP2-13657) (1:100), and HsSAS (Santa Cruz Biotechnology, Cat#SC-81431) (1:100). Cells were again washed with 1X PBS and incubated with Alexa fluor-488 anti-rabbit (Invitrogen, Cat#A21206) (1:500), Alexa fluor-488 anti-mouse (Invitrogen, Cat#A32766), Alexa fluor-568 anti-rabbit (Invitrogen, Cat#A10042), and Alexa fluor-568 anti-rat (Invitrogen, Cat#A11077) for 90 min at RT and washed before mounting with DAPI (LifeSpan Biosciences, Cat#LS-J1033-10) and imaged using a Zeiss confocal microscope at a magnification of ×63. We present images of ECs containing cilia in similar confluence areas on the plate. Primary cilia quantification for cilia size or length was measured using ACDC v0.93 cilia-specific software as described in our previous publication (Thirugnanam et al., 2022). For increasing cilia length in HBMECs, we incubated the cells with PDGF-BB ligand (10 ng/mL) for 60 min before staining. Briefly, cells were seeded in a six-well plate with a coverslip on it. After 24 h, the cells were changed to serum-free medium for contact inhibition and then changed to complete medium and treated with MNK2 inhibitor IV (Sigma, Cat#531206001) (2 μM for 24 h) to arrest the cells at the G0/G1 phase of the cell cycle. PDGF-BB ligand stimulation was performed, and then cells were subjected to staining with ARL13B cilia and CENTRIN 2 centrioles antibodies and imaged at ×100 oil immersion objective using a Keyence BZ-×700 fluorescent microscope (Japan). Texas Red filter cube (OP-87765, Keyence), a GFP filter cube (OP-87763, Keyence), and a 4′,6-diamidino-2-phenylindole (DAPI) filter cube (OP-87762, Keyence) were used to image the DAPI-stained samples.

### Primary brain ECs transfection

HBMECs were seeded in six-well culture dishes with (for IF) and without coverslip (for Western blot) approximately 24 h prior to transfection. *Control* and *CEP164 siRNA* (Horizon inspired cell solutions, Cat#D-001320–10–05, and Cat#J-020351-17-0005) were transfected using Lipofectamine 2000 reagent (Gibco, Cat#11668019) and incubated for 48 h. Cells were washed twice with PBS, replaced with a complete growth medium, and incubated at 37°C until further experimentation such as primary cilia immunostaining or Western blot.

### Fluorescence-activated cell sorting (FACS)

Cells were harvested from the six-well plates using TrypLE Express (Gibco, Cat#12604021). Single-cell suspensions were washed twice with FACS buffer (1× PBS with 5% FBS and 0.1% NaN_3_) at 300 g for 5 min. Then, cells were fixed and permeabilized using TF fix/perm buffer of a transcription factor buffer set (BD biosciences, Cat#562574) per the manufacturer’s protocol. Cells were stained for the following proteins: ARL13B, cyclin B1, cyclin A, cdc2 (CDK1), and ki67 (eBioscience, cat#25-5698-80). Suitable secondary reagents were used. Primary antibodies were diluted at 1:50 and secondary antibodies at 1:500. Following each staining, cells were washed thrice in 500 μL buffer using BD biosciences transcription factor (BD TF) perm wash buffer (component from Cat#562574). Primary antibodies were incubated for 45 min and secondaries for 30 min at 4°C with appropriate secondary controls. After staining, cells were resuspended in FACS buffer. For cell cycle analysis, per the manufacturer’s protocol, FxCycle Violet stain (Invitrogen, Cat#F10347) was added to cell suspensions just before running the samples in the flow cytometer (BD LSRFortessa). Sample acquisition was done using FACSDiva software (BD) with subsequent analysis on FlowJo software.

### Statistical analysis

Data were presented as the mean and standard error of the mean (SEM). A *t*-test, Welch’s *t*-test, or analysis of variance (ANOVA) was performed to compare the outcome measures between different groups or phases of the cell cycle. Cilia length from the mother and daughter centrioles of HBMECs was compared using the paired *t*-test. Count data were compared using a generalized linear model with a negative binomial distribution. For some analyses, data were log-transformed to improve fit. *p* < 0.05 was considered significant. Dunnett’s test was used to adjust for multiple comparisons. Statistical analysis was performed using SAS V9.4 (SAS Institute Inc., Cary, NC) and GraphPad Prism software.

## Results

### Brain microvascular endothelial cells possess more than one cilium, and the second cilium arises from the daughter centriole

**FIGURE 1:**
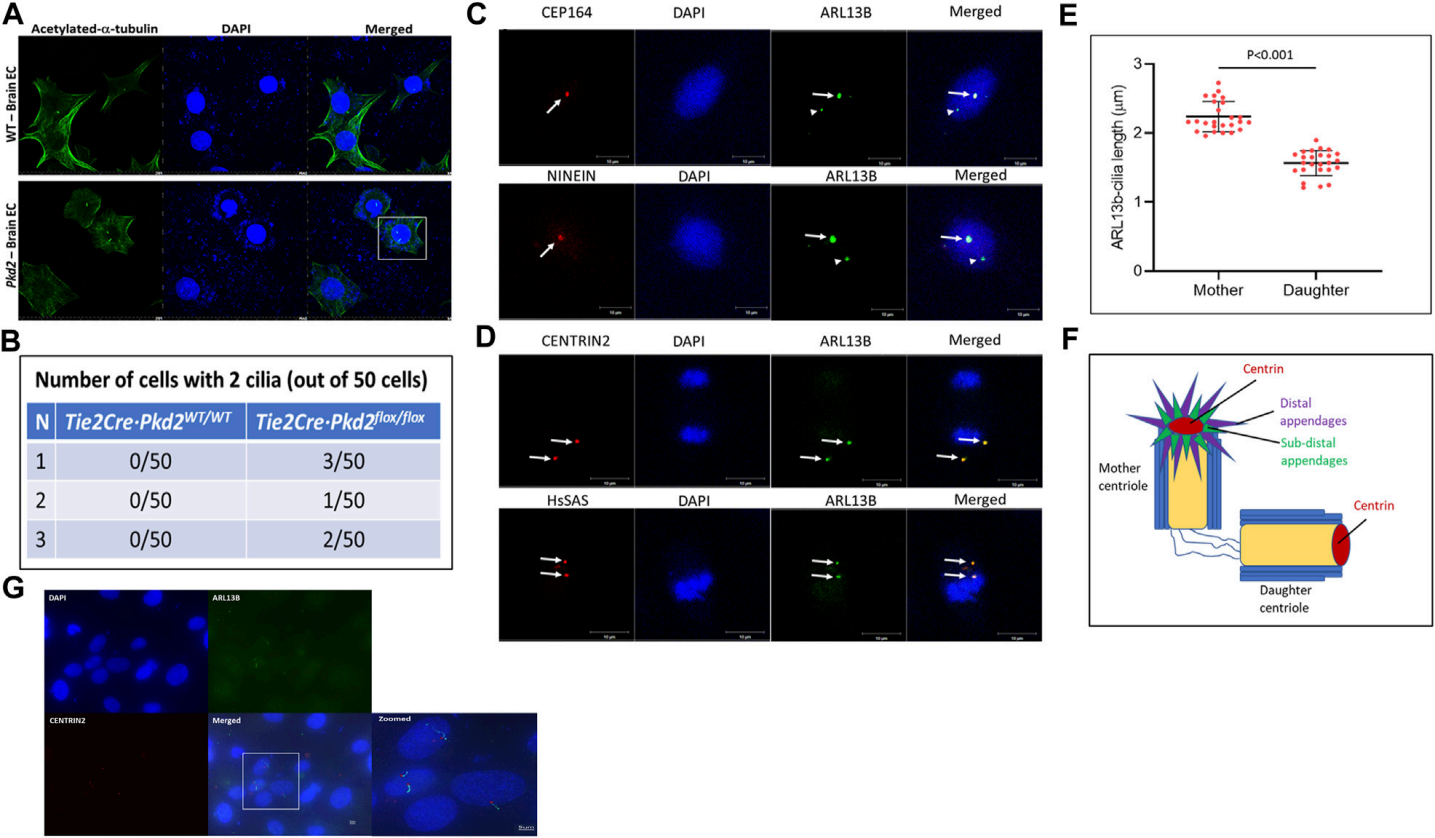
Brain ECs possess a second cilium from the daughter centriole. **(A)**. Brain endothelial cells from frontal cortex of one-week-old *Tie2Cre·Pkd2* ^*WT/WT*^ (with Cre activation; control wild-type group) and *Tie2Cre·Pkd2* ^*flox/flox*^ (with Cre activation; experimental *Pkd2* group) mice were collected and immunostained for primary cilia with acetylated-α-tubulin and nuclei with DAPI using the immunofluorescence method. Scale bar = 20 μm. **(B)**. The table represents the number of brain endothelial cells with a two-cilia phenotype. A minimum of 50 cells were assessed in triplicates for two-cilia phenotype detection. Scale bar = 10 μm. **(C)**. Human primary brain microvascular endothelial cells (HBMECs) were immunostained for mother centriole distal and subdistal appendage markers, CEP164 and NINEIN, respectively (red), nuclei stained with DAPI, and primary cilia stained with ARL13B (green). White arrows indicate cilia from the mother centriole and arrowheads represent second cilia from the daughter centriole. **(D)**. HBMECs stained for Centrin2 and HsSAS, markers for distal lumen and procentriolar markers of the mother and daughter centriole (Red), nuclei stained with DAPI, and primary cilia stained with ARL13B (green). White arrows indicate cilia from the mother and daughter centriole and their respective cilia. Scale bar = 10 μm. **(E)**. Primary cilia size varies based on ciliogenesis from the mother and daughter centrioles of HBMECs. N = 25 double cilia cells were quantified for their respective size. **(F)**. Pictorial representation of the centriole markers used in the study. All the experiments were performed in triplicates. Results are presented as mean ± SEM. SEM, standard error to the mean. *p* < 0.05 was considered significant. Statistics were performed using paired t-tests. **(G)**. HBMECs were contact inhibited, arrested at the G0/G1 phase of the cell cycle for 24 h, treated with PDGF-BB ligand for 60 min, immunostained for ARL13B cilia (green) and CENTRIN 2 (red) centriole antibodies, and imaged at 100X using a Keyence BZ-X700 fluorescent microscope (Japan). Scale bar = 5 μm.

Previously, we reported that HBMECs and human embryonic stem cell-derived brain microvascular ECs showed a two-cilia phenotype (Thirugnanam et al., 2022) *in vitro*. To investigate whether the two-cilia phenotype is observed in other conditions, we chose to investigate cilia in mouse primary brain ECs from polycystic kidney disease (*Pkd2)* knockout mouse, a ciliopathic condition associated with an abnormal primary ciliary function that causes phenotype of polyploidy and a defective cell cycle (AbouAlaiwi et al., 2011). We isolated brain ECs from the frontal cortices of *Tie2Cre·Pkd2* ^*WT/WT*^ (wild type; *WT*) and *Tie2Cre·Pkd2* ^*flox/flox*^ *(Pkd2)* mice. Isolated brain ECs were stained for ciliary marker acetylated-α-tubulin and nuclear marker DAPI by IF (Figure 1A). IF staining for ciliary markers suggested brain ECs isolated from *Pkd2* knockout mice possess more than one cilium. Approximately 1% of *Pkd2* null ECs possess two cilia (Figure 1B). To determine the origin of second cilium in brain ECs, we used HBMECs, and immunostained for co-localization of the primary cilia and centrioles. HBMECs were characterized extensively in our previous work (Thirugnanam et al., 2022). The centriole markers were chosen with the rationale to distinguish mother and daughter centrioles (Figure 1F). CEP164 and NINEIN are specific markers for distal and subdistal appendage regions of the mother centriole, respectively (Hall and Hehnly, 2021). Immunostaining mother centriole markers with ciliary axonemal markers ARL13B (Figure 1C) or IFT88 (Supplementary Figure S2) suggests only one cilium is co-localized with mother centriole markers CEP164 and NINEIN. We quantified the size of the cilia using an automated cilia-specific software ACDC v0.93 as described in our previous publication (Thirugnanam et al., 2022). The cilium arising from the mother centriole is bigger in size than the cilium from the daughter centriole (Figure 1E). Because the cilia appeared as dots without clear definition of the axoneme and basal body structure, we rationalized that increasing cilia length will offer better resolution of the two-cilia structure. Based on a past publication (Thirugnanam et al., 2022), we treated HBMECs with Platelet-Derived Growth Factor-BB (PDGF-BB) ligand for 60 min to increase the length of the cilia and stained for CENTRIN2, DAPI, and ARL13B markers. A single brain EC with two cilia that has the classical basal body (Figure 1G, red stain) and axoneme (Figure 1G, green stain) was visible. Additionally, cells with single cilium were also observed in the field. These results collectively suggest that the second cilium arises from the daughter centriole. Collectively, these results suggest brain microvascular ECs possess two cilia, and the second cilium arises from the daughter centriole. We also used a second set of centriole markers: CENTRIN2 and HsSAS markers, which label distal lumen and procentriole respectively of both mother and daughter centrioles (Hall and Hehnly, 2021). Indeed, ARL13B co-stained with ciliary markers CENTRIN2 and HsSAS (Figure 1D). CENTRIN2 was also confirmed with a second ciliary Intraflagellar Transport 88 (IFT88) (Supplementary Figure S2).

### Ciliary protein signaling occurs predominantly in the G0/G1 and S phases of the cell cycle

**FIGURE 2:**
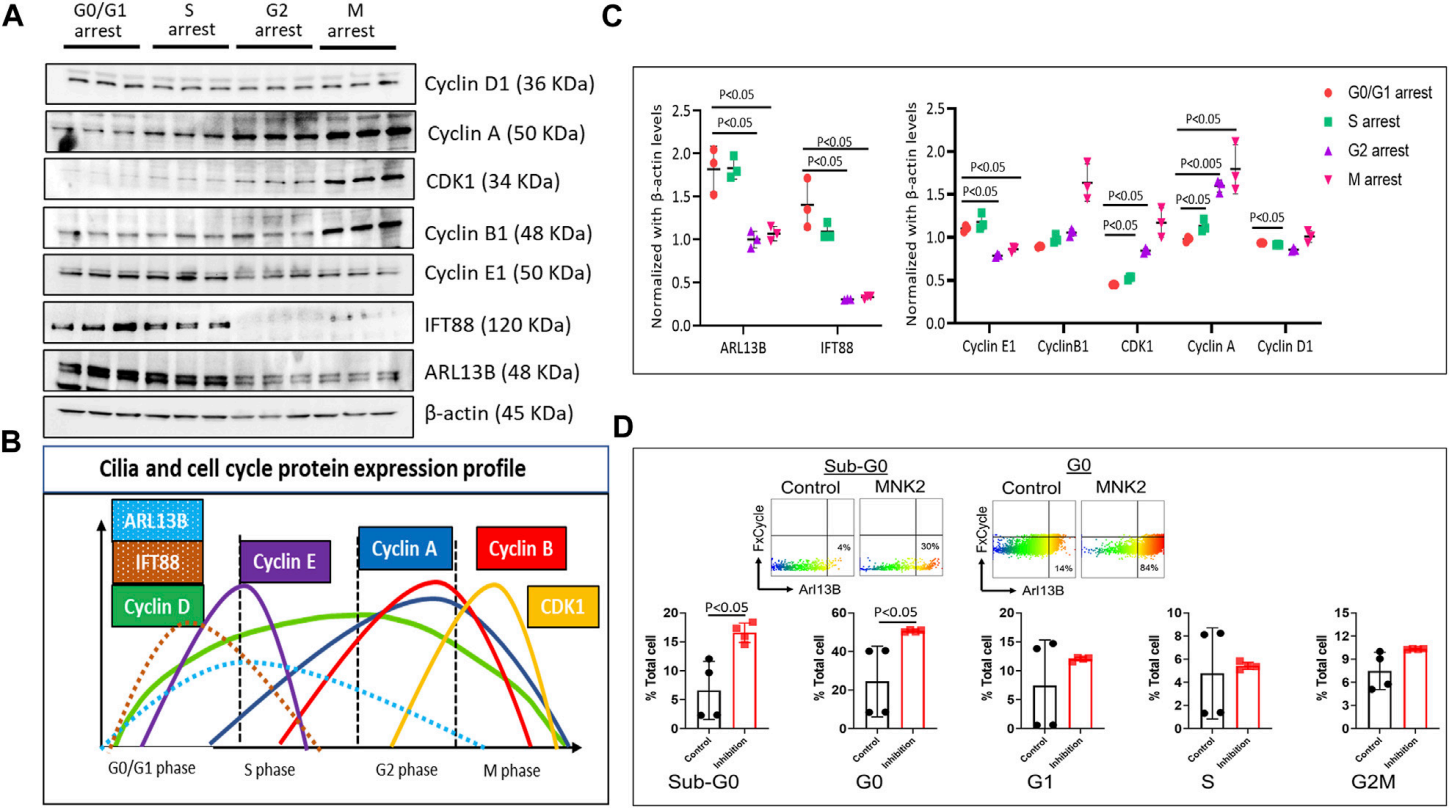
Cell cycle and ciliary protein expression profile. **(A)**. HBMECs were arrested at their respective cell cycle stages and assessed for cell cycle and ciliary proteins using the Western blotting method using MNK2 inhibitor at 2 μM for the G0/G1 phase, CI-994 at 10 μM for the S phase, MDM2 inhibitor at 100 nM for the G2 phase, and vinblastine sulfate at 2 nM for the M phase and **(B)**. quantified using Image J software and plotted in GraphPad prism software. **(C)**. Color-coded peaks represent the high and low ciliary and cell cycle protein expression in their respective cell cycle stage. *p* < 0.05 was considered significant; *n* = 3 in each experimental group. Results are presented as mean ± SEM. SEM, standard error to the mean. **(D)**. Quantification of ARL13B^Hi^ cells in various stages of the cell cycle. *p* < 0.05 was considered significant; *n* = 4 in each experimental group. Results are presented as mean ± SEM. SEM, standard error to the mean. ANOVA was used to examine the effects of various conditions on the outcomes.

During the cell cycle, ciliogenesis occurs at the G0/G1 phase. To assess cilia protein expression and regulation, we investigated the expression pattern of ciliary proteins ARL13B and IFT88 in different cell cycle phases. Briefly, HBMECs were arrested at different phases of the cell cycle using specific cell cycle inhibitory molecules: mitogen-activated protein kinase-interacting kinase 2 (MNK2) inhibitor (Yen et al., 2022) at 2 μM for G0/G1, CI-994 (an inhibitor of histone deacetylase 1) at 10 μM for the S phase, Murine Double Minute-2 (MDM2) inhibitor (which blocks p53 transcriptional activation) at 100 nM for the G2 phase, and vinblastine sulfate (a microtubule inhibitor) at 2 nM for the M phase. To confirm that the cells were arrested at different phases of the cell cycle, the expression profile of cyclin and CDK proteins were assessed using Western blot protein expression (Figures 2A, B). Cells arrested at different phases of the cell cycle revealed that ciliary proteins ARL13B and IFT88 expression were high only in the G0/G1 and S phases of the cell cycle (Figure 2C), suggesting that brain EC ciliogenesis occurs at the G0/G1 phase and extends into the S phase. The Western blot data were supported by flow cytometry analysis of the cell cycle (Supplementary Figure S3) and ciliary proteins. The net fluorescence intensity of ARL13B was highest in the G0/G1 phase (Supplementary Figure S3). Interestingly, in flow cytometry, we observed a fraction of cells distinctly high in ARL13B (ARL13B^Hi^) expression in the G0 and Sub-G0 cell cycle phases (Figure 2D). The number of ARL13B^Hi^ cells was even higher following G0/G1 arrest. At G0, ∼30% control cells displayed the ARL13B^Hi^ phenotype, while it was >40% following G0/G1 arrest (Figure 2D). These data collectively suggest that the ciliary proteins responsible for cilia growth and ciliary membrane trafficking (ARL13B) are synthesized during the G0/G1 phase of cell cycle.

### Brain endothelial cell two-cilia phenotype is specific to the G0/G1 phase of the cell cycle

**FIGURE 3:**
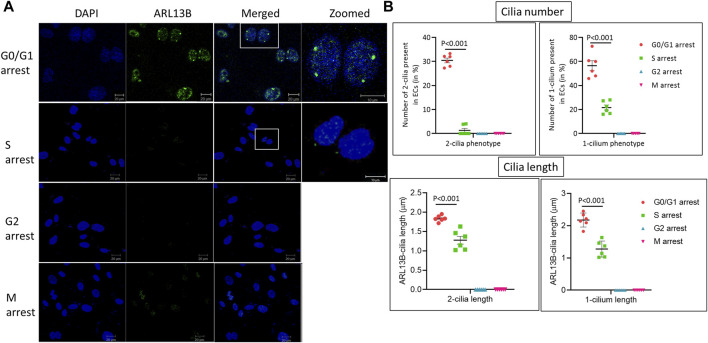
Human primary brain microvascular endothelial cells ciliogenesis at different cell cycle stages. **(A)**. HBMECs were arrested at the respective cell cycle stages using specific inhibitors, MNK2 inhibitor at 2 μM for the G0/G1 phase, CI-994 at 10 μM for the S phase, MDM2 inhibitor at 100 nM for the G2 phase and vinblastine sulfate at 2 nM for the M phase, and imaged using confocal microscopy for immunofluorescence. DAPI, ARL13B and merged images, scale bar = 10 μm and zoomed image, scale bar = 10 μm for zoomed. **(B)**. Quantification was done as mentioned in the methods section for cilia length and number. G0/G1 group, *n* = 154 nuclei; S group, *n* = 144 nuclei; G2 group, *n* = 143 nuclei; and M group, *n* = 153 nuclei. Statistical *p*-value comparisons across groups are shown (*n* = 6) for each quantification method. Results are presented as mean ± SEM. SEM, standard error to the mean. *p* < 0.05 was considered significant. Statistics were performed using paired t-tests.

To assess when in the cell cycle brain microvascular ECs exhibit the two-cilia phenotype, HBMECs were arrested at specific stages of the cell cycle using the cell cycle inhibitors mentioned above and analyzed using immunofluorescence (IF) with cilia-specific ARL13B antibody (Figure 3). In total, 30.6% of cells arrested at the G0/G1 phase exhibited the two-cilia phenotype and about 56.5% of the cells exhibited one cilium. Further, approximately 21.6% of the cells in the S phase exhibited one cilium, and about 1.3% of cells showed the two-cilia phenotype. We did not observe any one- or two-cilia phenotype in the G2/M phase. Taken together, these data suggest that brain ECs exhibiting the two-cilia phenotype are restricted to the G0/G1 phase of the cell cycle.

### CEP164-mediated mother centriole is required for cilia from the daughter centriole

**FIGURE 4:**
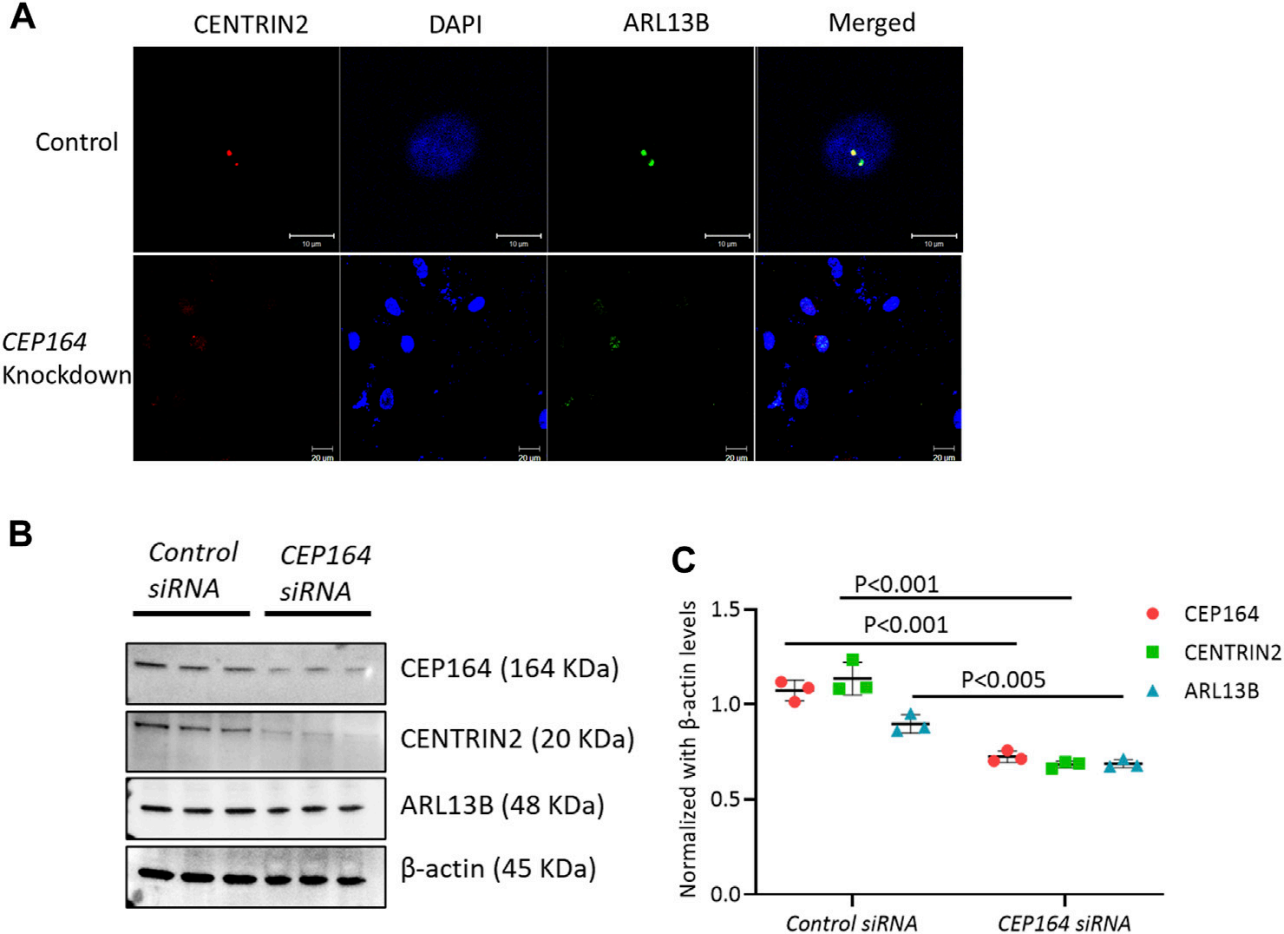
*CEP164* knockdown in HBMECs. **(A)**. HBMECs were knocked down for *CEP164 siRNA* and *control siRNA* and immunostained for CENTRIN2, a centriole antibody. **(B)**. *CEP164* knockdown was performed in HBMECs and protein expression of CEP164 for knockdown efficiency and CENTRIN2 and ARL13B was performed. **(C)**. Quantification of the Western blot was performed using ImageJ software. *n* = 3 in each experimental group. Results are presented as mean ± SEM. SEM, standard error to the mean. Statistics were performed using paired t-tests.

To assess whether ciliogenesis occurs from the daughter centriole, we knocked down (KD) *CEP164*. The regulation of CEP164 is essential for the proper recruitment of mother centriole-mediated membrane-associated ciliary proteins and primary ciliogenesis (Kobayashi et al., 2020). IF analysis of efficacy confirmed *CEP164* KD brain ECs showed minimal ciliogenesis from both the mother and daughter centriole (Figure 4A). Further, the protein expression of CENTRIN2, and ciliary proteins ARL13B were also depleted in the *CEP164* KD cells (Figures 4B, C). Taken together, these results suggest the role of the mother centriole is important in determining primary as well as secondary cilium from the daughter centriole in brain ECs.

## Discussion

In our recent study, we have shown that brain endothelial ciliogenesis is important in establishing vascular stability in the brain (Thirugnanam et al., 2022). In addition, we observed that brain ECs display more than one cilium. Cells possessing multiple primary cilia have been shown previously. For example, mouse brain collateral ECs and human islet beta cells of the pancreas possess a rare two-cilia phenotype and occasional a third cilia (Zhang et al., 2019; Polino et al., 2023). In our results, we also showed that brain ECs isolated from *Pkd2* ^*−/−*^ mice exhibit two cilia (Figure 1A). However, it is not clear when and how brain ECs exhibit two cilia and the importance of the second cilium. In this brief report, we investigated the first question of how and when brain ECs’ second cilia are formed. Our main finding is that the second cilium arises from the daughter centriole in brain ECs, and in the G0/G1 phase, which was not known previously.

The assembly of primary cilia is a tightly regulated, multistep process where the centriole plays a significant role in the onset of ciliogenesis. Both distal and subdistal appendages of the mother centriole have been primarily linked to functions in primary cilia formation (Hall and Hehnly, 2021). Knockdown or loss of distal appendage protein CEP164 has been shown to have defective primary ciliogenesis (Kobayashi et al., 2020) or cell cycle dysfunction (Slaats et al., 2014). Our results revealed that there is more than one cilium co-stained for mother centriole CEP164 or NINEIN (Figure 1C). The role of the mother centriole is well known in ciliogenesis. However, the role of the daughter centriole in ciliogenesis is poorly understood. A recent study implicates the role of the daughter centriole in primary cilia formation and suggest the proximity of the daughter centriole in determining ciliogenesis from the mother centriole (Loukil et al., 2017). Our IF data in ECs suggest that the second cilium arises from the daughter centriole. In addition, the second cilium is also different in terms of its size and is shorter compared with the cilium arising from the mother centriole (Figure 1E). However, they appeared as dots rather than ciliary structures with an axoneme and basal body. To ensure that the ciliary dot-like structures were indeed cilium, we decided to increase cilia length by ligand treatment such as PDGF-BB, which we previously reported to trigger ciliogenesis in brain ECs (Thirugnanam et al., 2022). Indeed, in PDGF-BB treated ECs, we observed (Figure 1G) ECs with two cilia and each cilium showed ciliary axoneme and basal body morphologies, which were distinct. To investigate the origin of the second cilium, we knocked down CEP164, a mother centriole marker, and found that the daughter centriole-second cilium is dependent on the mother centriole presence (Figure 4). The daughter centriole is implicated in motile cilia formation in multiciliated cells (Al Jord et al., 2014). Therefore, whether the brain EC second cilium is a hybrid cilium that was recently discovered in multiciliated mammalian cells (Liu et al., 2020) is not known. Studies in the spatial arrangement of microtubules in brain EC cilia is necessary. Electron microscopy or super-resolution microscopy and focused ion beam scanning electron microscopy imaging is needed and is part of the ongoing studies in our lab.

Primary ciliogenesis assembly and disassembly are tightly associated with cell cycle regulation. HBMECs arrested at specific stages of the cell cycle revealed that ciliary proteins ARL13B and IFT88 were predominantly expressed in the G0/G1 and S phases of the cell cycle (Figure 2). ARL13B, a small GTPase, is both a signaling protein and a marker for cilia (Larkins et al., 2011). In our previous study, we have shown that ARL13B overexpression in primary brain ECs induces ciliogenesis by increasing ciliary size and number (Thirugnanam et al., 2022). In the current study, using the FACS method, high ARL13B expressing ECs (ARL13B^Hi^) were found mostly in the G0 phase of the cell cycle (Figure 2D). Taken together, higher ARL13B expression level during the G0 phase is associated with brain ECs’ propensity to display multiple cilia. This interpretation partly explains why we observed a high number of ECs (30%) showing a two-cilia phenotype in the G0/G1 phase compared with the other cell cycle stages (Figure 3). In terms of other phases of the cell cycle, the two-cilia phenotype was noticed in a small fraction (∼1.3%) of ECs in the S phase, and no two-cilia phenotype was observed in the G2/M phase. In the S phase, residual levels fo ARL13B protein and in turn, its signaling activity may explain the small fraction of cells with 2-cilia, a hypothesis that needs further testing. In the G2/M phase, we have not observed a two-cilia or one-cilium phenotype. Cilia resorption usually occurs from the S phase and is completely resorbed in the M phase (Kim and Tsiokas, 2011). However, we cannot fully exclude the possibility of cilia presence in the G2/M phase in brain ECs because the size of brain EC cilium is relatively small (1–2 μm) compared to other primary cilia (HUVECs: >3 μm or primary kidney cells: ∼7 μm). The obvious next question is what is the purpose of the two-cilia phenotype in the G0/G1 phase? Because we did not observe two cilia in the normal state of cells and only during polyploidy-inducing conditions (*Pkd2* null ECs) or during the G0/G1 phase of arrested human primary brain ECs, and in a small fraction of cells (<5%), we hypothesize that the two-cilia cells may mark a defective state of the cell and are thus marked for triage. The significance of the brain EC two-cilia phenotype remains an open question and is subject of ongoing investigation in the laboratory.

In summary, in primary brain microvascular ECs, the two-cilia phenotype is found predominantly in the G0/G1 phase of the cell cycle. The second cilium originates from the daughter centriole and is dependent on the presence of the mother centriole.

## Data Availability

The original contributions presented in the study are included in the article/Supplementary Material, further inquiries can be directed to the corresponding author.
