# Supplementary material for: Brain microvascular endothelial cells possess a second cilium that arises from the daughter centriole
Source: Front Mol Biosci. 2023 Nov 6;10:1250016. doi: 10.3389/fmolb.2023.1250016 (PMC10657992; doi:10.3389/fmolb.2023.1250016)
Supplement: Supplementary file 1 [file Table1.docx]

# Manuscript figures detailed statistics

# Figure 1E

Paired t test was used.

Dif = mother – daughter of the same pair of centrioles within a cell

| **Variable** | **N** | **N Miss** | **Minimum** | **Mean** | **Std Dev** | **Median** | **Lower Quartile** | **Upper Quartile** | **Maximum** |
| --- | --- | --- | --- | --- | --- | --- | --- | --- | --- |
| \| **Mother** \| \| --- \| \| **Daughter** \| \| **dif** \| | \| 25 \| \| --- \| \| 25 \| \| 25 \| | \| 0 \| \| --- \| \| 0 \| \| 0 \| | \| 1.96 \| \| --- \| \| 1.21 \| \| 0.12 \| | \| 2.24 \| \| --- \| \| 1.56 \| \| 0.67 \| | \| 0.22 \| \| --- \| \| 0.18 \| \| 0.32 \| | \| 2.16 \| \| --- \| \| 1.57 \| \| 0.61 \| | \| 2.10 \| \| --- \| \| 1.47 \| \| 0.49 \| | \| 2.44 \| \| --- \| \| 1.69 \| \| 0.76 \| | \| 2.73 \| \| --- \| \| 1.90 \| \| 1.32 \| |

| **Tests for Location: Mu0=0** | | | | |
| --- | --- | --- | --- | --- |
| **Test** | **Statistic** | | **p Value** | |
| **Student's t** | **t** | 10.67912 | **Pr > \|t\|** | <.0001 |

# Figure 2C

## S phase vs G0/G1 phase

|  | | **N** | **NMiss** | **Min** | **Mean** | **Std** | **Median** | **Q1** | **Q3** | **Max** |
| --- | --- | --- | --- | --- | --- | --- | --- | --- | --- | --- |
| **ARl13B** | **G0/G1 Phase** | 3 | 0 | 1.52 | 1.82 | 0.27 | 1.89 | 1.52 | 2.05 | 2.05 |
|  | **S Phase** | 3 | 0 | 1.73 | 1.83 | 0.13 | 1.79 | 1.73 | 1.98 | 1.98 |

P=0.946

|  | | **N** | **NMiss** | **Min** | **Mean** | **Std** | **Median** | **Q1** | **Q3** | **Max** |
| --- | --- | --- | --- | --- | --- | --- | --- | --- | --- | --- |
| **IFT88** | **G0/G1 Phase** | 3 | 0 | 1.15 | 1.36 | 0.22 | 1.35 | 1.15 | 1.58 | 1.58 |
|  | **S Phase** | 3 | 0 | 1.04 | 1.09 | 0.09 | 1.04 | 1.04 | 1.20 | 1.20 |

P=0.119

|  | | **N** | **NMiss** | **Min** | **Mean** | **Std** | **Median** | **Q1** | **Q3** | **Max** |
| --- | --- | --- | --- | --- | --- | --- | --- | --- | --- | --- |
| **Cyclin_E1** | **G0/G1 Phase** | 3 | 0 | 1.06 | 1.10 | 0.04 | 1.11 | 1.06 | 1.14 | 1.14 |
|  | **S Phase** | 3 | 0 | 1.11 | 1.18 | 0.09 | 1.15 | 1.11 | 1.29 | 1.29 |

P=0.258

|  | | **N** | **NMiss** | **Min** | **Mean** | **Std** | **Median** | **Q1** | **Q3** | **Max** |
| --- | --- | --- | --- | --- | --- | --- | --- | --- | --- | --- |
| **CyclinB1** | **G0/G1 Phase** | 3 | 0 | 0.88 | 0.89 | 0.01 | 0.89 | 0.88 | 0.90 | 0.90 |
|  | **S Phase** | 3 | 0 | 0.88 | 0.92 | 0.04 | 0.94 | 0.88 | 0.94 | 0.94 |

P=0.277

|  | | **N** | **NMiss** | **Min** | **Mean** | **Std** | **Median** | **Q1** | **Q3** | **Max** |
| --- | --- | --- | --- | --- | --- | --- | --- | --- | --- | --- |
| **CDK1** | **G0/G1 Phase** | 3 | 0 | 0.82 | 0.83 | 0.01 | 0.83 | 0.82 | 0.84 | 0.84 |
|  | **S Phase** | 3 | 0 | 0.86 | 0.86 | 0.00 | 0.86 | 0.86 | 0.87 | 0.87 |

P=0.0057

|  | | **N** | **NMiss** | **Min** | **Mean** | **Std** | **Median** | **Q1** | **Q3** | **Max** |
| --- | --- | --- | --- | --- | --- | --- | --- | --- | --- | --- |
| **Cyclin_A** | **G0/G1 Phase** | 3 | 0 | 0.95 | 0.98 | 0.03 | 0.98 | 0.95 | 1.00 | 1.00 |
|  | **S Phase** | 3 | 0 | 1.08 | 1.13 | 0.07 | 1.11 | 1.08 | 1.21 | 1.21 |

P=0.024

|  | | **N** | **NMiss** | **Min** | **Mean** | **Std** | **Median** | **Q1** | **Q3** | **Max** |
| --- | --- | --- | --- | --- | --- | --- | --- | --- | --- | --- |
| **Cyclin_D1** | **G0/G1 Phase** | 3 | 0 | 0.93 | 0.94 | 0.00 | 0.93 | 0.93 | 0.94 | 0.94 |
|  | **S Phase** | 3 | 0 | 0.91 | 0.91 | 0.00 | 0.91 | 0.91 | 0.92 | 0.92 |

P=0024

## G2 phase vs G0/G1 phase

|  | | **N** | **NMiss** | **Min** | **Mean** | **Std** | **Median** | **Q1** | **Q3** | **Max** |
| --- | --- | --- | --- | --- | --- | --- | --- | --- | --- | --- |
| **ARl13B** | **G0/G1 Phase** | 3 | 0 | 1.52 | 1.82 | 0.27 | 1.89 | 1.52 | 2.05 | 2.05 |
|  | **G2 Phase** | 3 | 0 | 1.00 | 1.11 | 0.11 | 1.11 | 1.00 | 1.22 | 1.22 |

P=0.013

|  | | **N** | **NMiss** | **Min** | **Mean** | **Std** | **Median** | **Q1** | **Q3** | **Max** |
| --- | --- | --- | --- | --- | --- | --- | --- | --- | --- | --- |
| **IFT88** | **G0/G1 Phase** | 3 | 0 | 1.15 | 1.36 | 0.22 | 1.35 | 1.15 | 1.58 | 1.58 |
|  | **G2 Phase** | 3 | 0 | 0.79 | 0.82 | 0.03 | 0.82 | 0.79 | 0.85 | 0.85 |

P=0.047

|  | | **N** | **NMiss** | **Min** | **Mean** | **Std** | **Median** | **Q1** | **Q3** | **Max** |
| --- | --- | --- | --- | --- | --- | --- | --- | --- | --- | --- |
| **Cyclin_E1** | **G0/G1 Phase** | 3 | 0 | 1.06 | 1.10 | 0.04 | 1.11 | 1.06 | 1.14 | 1.14 |
|  | **G2 Phase** | 3 | 0 | 0.94 | 0.98 | 0.04 | 0.98 | 0.94 | 1.01 | 1.01 |

P=0.013

|  | | **N** | **NMiss** | **Min** | **Mean** | **Std** | **Median** | **Q1** | **Q3** | **Max** |
| --- | --- | --- | --- | --- | --- | --- | --- | --- | --- | --- |
| **CyclinB1** | **G0/G1 Phase** | 3 | 0 | 0.88 | 0.89 | 0.01 | 0.89 | 0.88 | 0.90 | 0.90 |
|  | **G2 Phase** | 3 | 0 | 0.93 | 0.98 | 0.07 | 0.96 | 0.93 | 1.06 | 1.06 |

P=0.14

|  | | **N** | **NMiss** | **Min** | **Mean** | **Std** | **Median** | **Q1** | **Q3** | **Max** |
| --- | --- | --- | --- | --- | --- | --- | --- | --- | --- | --- |
| **CDK1** | **G0/G1 Phase** | 3 | 0 | 0.82 | 0.83 | 0.01 | 0.83 | 0.82 | 0.84 | 0.84 |
|  | **G2 Phase** | 3 | 0 | 0.91 | 0.94 | 0.03 | 0.94 | 0.91 | 0.97 | 0.97 |

P=0.0039

|  | | **N** | **NMiss** | **Min** | **Mean** | **Std** | **Median** | **Q1** | **Q3** | **Max** |
| --- | --- | --- | --- | --- | --- | --- | --- | --- | --- | --- |
| **Cyclin_A** | **G0/G1 Phase** | 3 | 0 | 0.95 | 0.98 | 0.03 | 0.98 | 0.95 | 1.00 | 1.00 |
|  | **G2 Phase** | 3 | 0 | 1.44 | 1.51 | 0.06 | 1.53 | 1.44 | 1.55 | 1.55 |

P=0.0001

|  | | **N** | **NMiss** | **Min** | **Mean** | **Std** | **Median** | **Q1** | **Q3** | **Max** |
| --- | --- | --- | --- | --- | --- | --- | --- | --- | --- | --- |
| **Cyclin_D1** | **G0/G1 Phase** | 3 | 0 | 0.93 | 0.94 | 0.00 | 0.93 | 0.93 | 0.94 | 0.94 |
|  | **G2 Phase** | 3 | 0 | 0.89 | 0.91 | 0.02 | 0.90 | 0.89 | 0.93 | 0.93 |

P=0.11

## M phase vs G0/G1 phase

|  | | **N** | **NMiss** | **Min** | **Mean** | **Std** | **Median** | **Q1** | **Q3** | **Max** |
| --- | --- | --- | --- | --- | --- | --- | --- | --- | --- | --- |
| **ARl13B** | **G0/G1 Phase** | 3 | 0 | 1.52 | 1.82 | 0.27 | 1.89 | 1.52 | 2.05 | 2.05 |
|  | **M Phase** | 3 | 0 | 1.04 | 1.09 | 0.06 | 1.07 | 1.04 | 1.15 | 1.15 |

P=0.010

|  | | **N** | **NMiss** | **Min** | **Mean** | **Std** | **Median** | **Q1** | **Q3** | **Max** |
| --- | --- | --- | --- | --- | --- | --- | --- | --- | --- | --- |
| **IFT88** | **G0/G1 Phase** | 3 | 0 | 1.15 | 1.36 | 0.22 | 1.35 | 1.15 | 1.58 | 1.58 |
|  | **M Phase** | 3 | 0 | 0.79 | 0.82 | 0.02 | 0.82 | 0.79 | 0.84 | 0.84 |

P=0.047

|  | | **N** | **NMiss** | **Min** | **Mean** | **Std** | **Median** | **Q1** | **Q3** | **Max** |
| --- | --- | --- | --- | --- | --- | --- | --- | --- | --- | --- |
| **Cyclin_E1** | **G0/G1 Phase** | 3 | 0 | 1.06 | 1.10 | 0.04 | 1.11 | 1.06 | 1.14 | 1.14 |
|  | **M Phase** | 3 | 0 | 0.96 | 0.96 | 0.01 | 0.96 | 0.96 | 0.97 | 0.97 |

P=0.020

|  | | **N** | **NMiss** | **Min** | **Mean** | **Std** | **Median** | **Q1** | **Q3** | **Max** |
| --- | --- | --- | --- | --- | --- | --- | --- | --- | --- | --- |
| **CyclinB1** | **G0/G1 Phase** | 3 | 0 | 0.88 | 0.89 | 0.01 | 0.89 | 0.88 | 0.90 | 0.90 |
|  | **M Phase** | 3 | 0 | 1.10 | 1.32 | 0.25 | 1.28 | 1.10 | 1.59 | 1.59 |

P=0.095

|  | | **N** | **NMiss** | **Min** | **Mean** | **Std** | **Median** | **Q1** | **Q3** | **Max** |
| --- | --- | --- | --- | --- | --- | --- | --- | --- | --- | --- |
| **CDK1** | **G0/G1 Phase** | 3 | 0 | 0.82 | 0.83 | 0.01 | 0.83 | 0.82 | 0.84 | 0.84 |
|  | **M Phase** | 3 | 0 | 1.06 | 1.19 | 0.15 | 1.17 | 1.06 | 1.34 | 1.34 |

P=0.0501

|  | | **N** | **NMiss** | **Min** | **Mean** | **Std** | **Median** | **Q1** | **Q3** | **Max** |
| --- | --- | --- | --- | --- | --- | --- | --- | --- | --- | --- |
| **Cyclin_A** | **G0/G1 Phase** | 3 | 0 | 0.95 | 0.98 | 0.03 | 0.98 | 0.95 | 1.00 | 1.00 |
|  | **M Phase** | 3 | 0 | 1.64 | 1.82 | 0.25 | 1.71 | 1.64 | 2.11 | 2.11 |

P=0.028

|  | | **N** | **NMiss** | **Min** | **Mean** | **Std** | **Median** | **Q1** | **Q3** | **Max** |
| --- | --- | --- | --- | --- | --- | --- | --- | --- | --- | --- |
| **Cyclin_D1** | **G0/G1 Phase** | 3 | 0 | 0.93 | 0.94 | 0.00 | 0.93 | 0.93 | 0.94 | 0.94 |
|  | **M Phase** | 3 | 0 | 0.90 | 0.93 | 0.04 | 0.92 | 0.90 | 0.97 | 0.97 |

P=0.86

# Figure 2D

## __Net_Arl13B_hi_in_sub_G0

|  | | **N** | **NMiss** | **Min** | **Mean** | **Std** | **Median** | **Q1** | **Q3** | **Max** |
| --- | --- | --- | --- | --- | --- | --- | --- | --- | --- | --- |
| **__Net_Arl13B_hi_in_sub_G0** | **Control** | 4 | 0 | 2.29 | 6.60 | 5.03 | 6.02 | 2.32 | 10.87 | 12.06 |
|  | **G0G1 inh** | 4 | 0 | 14.52 | 16.56 | 1.69 | 16.65 | 15.24 | 17.88 | 18.41 |

Variable: __Net_Arl13B_hi_in_sub_G0

| **Group** | **Method** | **N** | **Mean** | **Std Dev** | **Std Err** | **Minimum** | **Maximum** |
| --- | --- | --- | --- | --- | --- | --- | --- |
| **Control** |  | 4 | 6.5976 | 5.0300 | 2.5150 | 2.2919 | 12.0640 |
| **G0G1 inh** |  | 4 | 16.5593 | 1.6866 | 0.8433 | 14.5236 | 18.4080 |
| **Diff (1-2)** | **Pooled** |  | -9.9617 | 3.7513 | 2.6526 |  |  |
| **Diff (1-2)** | **Satterthwaite** |  | -9.9617 |  | 2.6526 |  |  |

| **Group** | **Method** | **Mean** | **95% CL Mean** | | **Std Dev** | **95% CL Std Dev** | |
| --- | --- | --- | --- | --- | --- | --- | --- |
| **Control** |  | 6.5976 | -1.4062 | 14.6014 | 5.0300 | 2.8494 | 18.7544 |
| **G0G1 inh** |  | 16.5593 | 13.8755 | 19.2430 | 1.6866 | 0.9554 | 6.2885 |
| **Diff (1-2)** | **Pooled** | -9.9617 | -16.4523 | -3.4710 | 3.7513 | 2.4173 | 8.2607 |
| **Diff (1-2)** | **Satterthwaite** | -9.9617 | -17.5986 | -2.3247 |  |  |  |

| **Method** | **Variances** | **DF** | **t Value** | **Pr > \|t\|** |
| --- | --- | --- | --- | --- |
| **Pooled** | Equal | 6 | -3.76 | 0.0094 |
| **Satterthwaite** | Unequal | 3.6662 | -3.76 | 0.0232 |

| **Equality of Variances** | | | | |
| --- | --- | --- | --- | --- |
| **Method** | **Num DF** | **Den DF** | **F Value** | **Pr > F** |
| **Folded F** | 3 | 3 | 8.89 | 0.1057 |

## __Net_Arl13B_hi_in_G0

|  | | **N** | **NMiss** | **Min** | **Mean** | **Std** | **Median** | **Q1** | **Q3** | **Max** |
| --- | --- | --- | --- | --- | --- | --- | --- | --- | --- | --- |
| **__Net_Arl13B_hi_in_G0** | **Control** | 4 | 0 | 8.47 | 24.42 | 18.34 | 24.38 | 8.54 | 40.30 | 40.45 |
|  | **G0G1 inh** | 4 | 0 | 50.10 | 50.61 | 0.57 | 50.58 | 50.12 | 51.10 | 51.20 |

Variable: __Net_Arl13B_hi_in_G0

| **Group** | **Method** | **N** | **Mean** | **Std Dev** | **Std Err** | **Minimum** | **Maximum** |
| --- | --- | --- | --- | --- | --- | --- | --- |
| **Control** |  | 4 | 24.4183 | 18.3353 | 9.1677 | 8.4711 | 40.4494 |
| **G0G1 inh** |  | 4 | 50.6129 | 0.5704 | 0.2852 | 50.0976 | 51.1964 |
| **Diff (1-2)** | **Pooled** |  | -26.1946 | 12.9713 | 9.1721 |  |  |
| **Diff (1-2)** | **Satterthwaite** |  | -26.1946 |  | 9.1721 |  |  |

| **Group** | **Method** | **Mean** | **95% CL Mean** | | **Std Dev** | **95% CL Std Dev** | |
| --- | --- | --- | --- | --- | --- | --- | --- |
| **Control** |  | 24.4183 | -4.7573 | 53.5939 | 18.3353 | 10.3868 | 68.3642 |
| **G0G1 inh** |  | 50.6129 | 49.7053 | 51.5204 | 0.5704 | 0.3231 | 2.1266 |
| **Diff (1-2)** | **Pooled** | -26.1946 | -48.6379 | -3.7512 | 12.9713 | 8.3586 | 28.5637 |
| **Diff (1-2)** | **Satterthwaite** | -26.1946 | -55.3524 | 2.9633 |  |  |  |

| **Method** | **Variances** | **DF** | **t Value** | **Pr > \|t\|** |
| --- | --- | --- | --- | --- |
| **Pooled** | Equal | 6 | -2.86 | 0.0290 |
| **Satterthwaite** | Unequal | 3.0058 | -2.86 | 0.0646 |

| **Equality of Variances** | | | | |
| --- | --- | --- | --- | --- |
| **Method** | **Num DF** | **Den DF** | **F Value** | **Pr > F** |
| **Folded F** | 3 | 3 | 1033.45 | 0.0001 |

## __Net_Arl13B_hi_in_G1

|  | | **N** | **NMiss** | **Min** | **Mean** | **Std** | **Median** | **Q1** | **Q3** | **Max** |
| --- | --- | --- | --- | --- | --- | --- | --- | --- | --- | --- |
| **__Net_Arl13B_hi_in_G1** | **Control** | 4 | 0 | 0.61 | 7.44 | 7.89 | 7.23 | 0.62 | 14.26 | 14.71 |
|  | **G0G1 inh** | 4 | 0 | 11.55 | 12.02 | 0.33 | 12.13 | 11.80 | 12.24 | 12.27 |

Variable: __Net_Arl13B_hi_in_G1

| **Group** | **Method** | **N** | **Mean** | **Std Dev** | **Std Err** | **Minimum** | **Maximum** |
| --- | --- | --- | --- | --- | --- | --- | --- |
| **Control** |  | 4 | 7.4425 | 7.8857 | 3.9429 | 0.6072 | 14.7056 |
| **G0G1 inh** |  | 4 | 12.0177 | 0.3280 | 0.1640 | 11.5455 | 12.2682 |
| **Diff (1-2)** | **Pooled** |  | -4.5752 | 5.5809 | 3.9463 |  |  |
| **Diff (1-2)** | **Satterthwaite** |  | -4.5752 |  | 3.9463 |  |  |

| **Group** | **Method** | **Mean** | **95% CL Mean** | | **Std Dev** | **95% CL Std Dev** | |
| --- | --- | --- | --- | --- | --- | --- | --- |
| **Control** |  | 7.4425 | -5.1055 | 19.9905 | 7.8857 | 4.4672 | 29.4024 |
| **G0G1 inh** |  | 12.0177 | 11.4958 | 12.5396 | 0.3280 | 0.1858 | 1.2229 |
| **Diff (1-2)** | **Pooled** | -4.5752 | -14.2313 | 5.0810 | 5.5809 | 3.5963 | 12.2895 |
| **Diff (1-2)** | **Satterthwaite** | -4.5752 | -17.1095 | 7.9592 |  |  |  |

| **Method** | **Variances** | **DF** | **t Value** | **Pr > \|t\|** |
| --- | --- | --- | --- | --- |
| **Pooled** | Equal | 6 | -1.16 | 0.2904 |
| **Satterthwaite** | Unequal | 3.0104 | -1.16 | 0.3299 |

| **Equality of Variances** | | | | |
| --- | --- | --- | --- | --- |
| **Method** | **Num DF** | **Den DF** | **F Value** | **Pr > F** |
| **Folded F** | 3 | 3 | 578.06 | 0.0002 |

## __Net_Arl13B_hi_in_S

|  | | **N** | **NMiss** | **Min** | **Mean** | **Std** | **Median** | **Q1** | **Q3** | **Max** |
| --- | --- | --- | --- | --- | --- | --- | --- | --- | --- | --- |
| **__Net_Arl13B_hi_in_S** | **Control** | 4 | 0 | 1.33 | 4.77 | 3.94 | 4.76 | 1.36 | 8.18 | 8.23 |
|  | **G0G1 inh** | 4 | 0 | 5.10 | 5.42 | 0.31 | 5.38 | 5.22 | 5.62 | 5.84 |

Variable: __Net_Arl13B_hi_in_S

| **Group** | **Method** | **N** | **Mean** | **Std Dev** | **Std Err** | **Minimum** | **Maximum** |
| --- | --- | --- | --- | --- | --- | --- | --- |
| **Control** |  | 4 | 4.7713 | 3.9353 | 1.9676 | 1.3320 | 8.2344 |
| **G0G1 inh** |  | 4 | 5.4240 | 0.3084 | 0.1542 | 5.0976 | 5.8404 |
| **Diff (1-2)** | **Pooled** |  | -0.6527 | 2.7912 | 1.9737 |  |  |
| **Diff (1-2)** | **Satterthwaite** |  | -0.6527 |  | 1.9737 |  |  |

| **Group** | **Method** | **Mean** | **95% CL Mean** | | **Std Dev** | **95% CL Std Dev** | |
| --- | --- | --- | --- | --- | --- | --- | --- |
| **Control** |  | 4.7713 | -1.4907 | 11.0332 | 3.9353 | 2.2293 | 14.6729 |
| **G0G1 inh** |  | 5.4240 | 4.9332 | 5.9148 | 0.3084 | 0.1747 | 1.1500 |
| **Diff (1-2)** | **Pooled** | -0.6527 | -5.4821 | 4.1767 | 2.7912 | 1.7986 | 6.1464 |
| **Diff (1-2)** | **Satterthwaite** | -0.6527 | -6.8909 | 5.5855 |  |  |  |

| **Method** | **Variances** | **DF** | **t Value** | **Pr > \|t\|** |
| --- | --- | --- | --- | --- |
| **Pooled** | Equal | 6 | -0.33 | 0.7521 |
| **Satterthwaite** | Unequal | 3.0369 | -0.33 | 0.7624 |

| **Equality of Variances** | | | | |
| --- | --- | --- | --- | --- |
| **Method** | **Num DF** | **Den DF** | **F Value** | **Pr > F** |
| **Folded F** | 3 | 3 | 162.78 | 0.0016 |

## __Net_Arl13B_hi_in_G2M

|  | | **N** | **NMiss** | **Min** | **Mean** | **Std** | **Median** | **Q1** | **Q3** | **Max** |
| --- | --- | --- | --- | --- | --- | --- | --- | --- | --- | --- |
| **__Net_Arl13B_hi_in_G2M** | **Control** | 4 | 0 | 5.09 | 7.47 | 2.41 | 7.41 | 5.42 | 9.53 | 9.99 |
|  | **G0G1 inh** | 4 | 0 | 10.23 | 10.31 | 0.07 | 10.32 | 10.25 | 10.38 | 10.38 |

Variable: __Net_Arl13B_hi_in_G2M

| **Group** | **Method** | **N** | **Mean** | **Std Dev** | **Std Err** | **Minimum** | **Maximum** |
| --- | --- | --- | --- | --- | --- | --- | --- |
| **Control** |  | 4 | 7.4746 | 2.4148 | 1.2074 | 5.0920 | 9.9946 |
| **G0G1 inh** |  | 4 | 10.3119 | 0.0748 | 0.0374 | 10.2312 | 10.3840 |
| **Diff (1-2)** | **Pooled** |  | -2.8373 | 1.7083 | 1.2080 |  |  |
| **Diff (1-2)** | **Satterthwaite** |  | -2.8373 |  | 1.2080 |  |  |

| **Group** | **Method** | **Mean** | **95% CL Mean** | | **Std Dev** | **95% CL Std Dev** | |
| --- | --- | --- | --- | --- | --- | --- | --- |
| **Control** |  | 7.4746 | 3.6321 | 11.3170 | 2.4148 | 1.3679 | 9.0036 |
| **G0G1 inh** |  | 10.3119 | 10.1929 | 10.4308 | 0.0748 | 0.0423 | 0.2787 |
| **Diff (1-2)** | **Pooled** | -2.8373 | -5.7931 | 0.1185 | 1.7083 | 1.1008 | 3.7619 |
| **Diff (1-2)** | **Satterthwaite** | -2.8373 | -6.6774 | 1.0028 |  |  |  |

| **Method** | **Variances** | **DF** | **t Value** | **Pr > \|t\|** |
| --- | --- | --- | --- | --- |
| **Pooled** | Equal | 6 | -2.35 | 0.0572 |
| **Satterthwaite** | Unequal | 3.0058 | -2.35 | 0.1002 |

| **Equality of Variances** | | | | |
| --- | --- | --- | --- | --- |
| **Method** | **Num DF** | **Den DF** | **F Value** | **Pr > F** |
| **Folded F** | 3 | 3 | 1043.38 | 0.0001 |

# Figure 3B (G0/G1 vs S)

## 2-cilia

### Percent Cilia (%)

|  | | **N** | **NMiss** | **Min** | **Mean** | **Std** | **Median** | **Q1** | **Q3** | **Max** |
| --- | --- | --- | --- | --- | --- | --- | --- | --- | --- | --- |
| **Percent_Cilia___** | **G0/G1** | 6 | 0 | 27.27 | 30.46 | 2.44 | 31.00 | 28.00 | 32.14 | 33.33 |
|  | **S** | 6 | 0 | 0.00 | 1.31 | 2.03 | 0.00 | 0.00 | 3.85 | 4.00 |

| **Image_Name** | **Method** | **N** | **Mean** | **Std Dev** | **Std Err** | **Minimum** | **Maximum** |
| --- | --- | --- | --- | --- | --- | --- | --- |
| **G0/G1** |  | 6 | 30.4582 | 2.4446 | 0.9980 | 27.2727 | 33.3333 |
| **S** |  | 6 | 1.3077 | 2.0265 | 0.8273 | 0 | 4.0000 |
| **Diff (1-2)** | **Pooled** |  | 29.1505 | 2.2453 | 1.2963 |  |  |
| **Diff (1-2)** | **Satterthwaite** |  | 29.1505 |  | 1.2963 |  |  |

| **Image_Name** | **Method** | **Mean** | **95% CL Mean** | | **Std Dev** | **95% CL Std Dev** | |
| --- | --- | --- | --- | --- | --- | --- | --- |
| **G0/G1** |  | 30.4582 | 27.8927 | 33.0237 | 2.4446 | 1.5260 | 5.9958 |
| **S** |  | 1.3077 | -0.8189 | 3.4343 | 2.0265 | 1.2649 | 4.9701 |
| **Diff (1-2)** | **Pooled** | 29.1505 | 26.2621 | 32.0389 | 2.2453 | 1.5688 | 3.9404 |
| **Diff (1-2)** | **Satterthwaite** | 29.1505 | 26.2485 | 32.0524 |  |  |  |

| **Method** | **Variances** | **DF** | **t Value** | **Pr > \|t\|** |
| --- | --- | --- | --- | --- |
| **Pooled** | Equal | 10 | 22.49 | <.0001 |
| **Satterthwaite** | Unequal | 9.6675 | 22.49 | <.0001 |

| **Equality of Variances** | | | | |
| --- | --- | --- | --- | --- |
| **Method** | **Num DF** | **Den DF** | **F Value** | **Pr > F** |
| **Folded F** | 5 | 5 | 1.46 | 0.6906 |

### Average Cilia Length (µm)

|  | | **N** | **NMiss** | **Min** | **Mean** | **Std** | **Median** | **Q1** | **Q3** | **Max** |
| --- | --- | --- | --- | --- | --- | --- | --- | --- | --- | --- |
| **VAR7** | **G0/G1** | 6 | 0 | 1.82 | 2.15 | 0.23 | 2.15 | 2.00 | 2.34 | 2.45 |
|  | **S** | 6 | 0 | 1.02 | 1.28 | 0.25 | 1.26 | 1.03 | 1.46 | 1.63 |

| **Image_Name** | **Method** | **N** | **Mean** | **Std Dev** | **Std Err** | **Minimum** | **Maximum** |
| --- | --- | --- | --- | --- | --- | --- | --- |
| **G0/G1** |  | 6 | 2.1530 | 0.2286 | 0.0933 | 1.8230 | 2.4513 |
| **S** |  | 6 | 1.2750 | 0.2499 | 0.1020 | 1.0192 | 1.6304 |
| **Diff (1-2)** | **Pooled** |  | 0.8780 | 0.2394 | 0.1382 |  |  |
| **Diff (1-2)** | **Satterthwaite** |  | 0.8780 |  | 0.1382 |  |  |

| **Image_Name** | **Method** | **Mean** | **95% CL Mean** | | **Std Dev** | **95% CL Std Dev** | |
| --- | --- | --- | --- | --- | --- | --- | --- |
| **G0/G1** |  | 2.1530 | 1.9132 | 2.3929 | 0.2286 | 0.1427 | 0.5606 |
| **S** |  | 1.2750 | 1.0128 | 1.5373 | 0.2499 | 0.1560 | 0.6128 |
| **Diff (1-2)** | **Pooled** | 0.8780 | 0.5700 | 1.1860 | 0.2394 | 0.1673 | 0.4202 |
| **Diff (1-2)** | **Satterthwaite** | 0.8780 | 0.5696 | 1.1864 |  |  |  |

| **Method** | **Variances** | **DF** | **t Value** | **Pr > \|t\|** |
| --- | --- | --- | --- | --- |
| **Pooled** | Equal | 10 | 6.35 | <.0001 |
| **Satterthwaite** | Unequal | 9.9217 | 6.35 | <.0001 |

| **Equality of Variances** | | | | |
| --- | --- | --- | --- | --- |
| **Method** | **Num DF** | **Den DF** | **F Value** | **Pr > F** |
| **Folded F** | 5 | 5 | 1.20 | 0.8497 |

## 1-cilia

### Percent Cilia (%)

|  | | **N** | **NMiss** | **Min** | **Mean** | **Std** | **Median** | **Q1** | **Q3** | **Max** |
| --- | --- | --- | --- | --- | --- | --- | --- | --- | --- | --- |
| **VAR9** | **G0/G1** | 6 | 0 | 45.83 | 56.55 | 10.00 | 56.00 | 48.00 | 60.71 | 72.73 |
|  | **S** | 6 | 0 | 16.00 | 21.65 | 5.21 | 20.98 | 16.67 | 27.27 | 28.00 |

| **Image_Name** | **Method** | **N** | **Mean** | **Std Dev** | **Std Err** | **Minimum** | **Maximum** |
| --- | --- | --- | --- | --- | --- | --- | --- |
| **G0/G1** |  | 6 | 56.5458 | 9.9960 | 4.0808 | 45.8333 | 72.7273 |
| **S** |  | 6 | 21.6496 | 5.2097 | 2.1269 | 16.0000 | 28.0000 |
| **Diff (1-2)** | **Pooled** |  | 34.8962 | 7.9706 | 4.6018 |  |  |
| **Diff (1-2)** | **Satterthwaite** |  | 34.8962 |  | 4.6018 |  |  |

| **Image_Name** | **Method** | **Mean** | **95% CL Mean** | | **Std Dev** | **95% CL Std Dev** | |
| --- | --- | --- | --- | --- | --- | --- | --- |
| **G0/G1** |  | 56.5458 | 46.0557 | 67.0360 | 9.9960 | 6.2396 | 24.5163 |
| **S** |  | 21.6496 | 16.1823 | 27.1169 | 5.2097 | 3.2520 | 12.7775 |
| **Diff (1-2)** | **Pooled** | 34.8962 | 24.6427 | 45.1498 | 7.9706 | 5.5692 | 13.9879 |
| **Diff (1-2)** | **Satterthwaite** | 34.8962 | 24.1679 | 45.6246 |  |  |  |

| **Method** | **Variances** | **DF** | **t Value** | **Pr > \|t\|** |
| --- | --- | --- | --- | --- |
| **Pooled** | Equal | 10 | 7.58 | <.0001 |
| **Satterthwaite** | Unequal | 7.5297 | 7.58 | <.0001 |

| **Equality of Variances** | | | | |
| --- | --- | --- | --- | --- |
| **Method** | **Num DF** | **Den DF** | **F Value** | **Pr > F** |
| **Folded F** | 5 | 5 | 3.68 | 0.1789 |

### Average Cilia Length (µm)

|  | | **N** | **NMiss** | **Min** | **Mean** | **Std** | **Median** | **Q1** | **Q3** | **Max** |
| --- | --- | --- | --- | --- | --- | --- | --- | --- | --- | --- |
| **VAR11** | **G0/G1** | 6 | 0 | 1.82 | 2.18 | 0.22 | 2.18 | 2.09 | 2.34 | 2.45 |
|  | **S** | 6 | 0 | 1.02 | 1.28 | 0.25 | 1.26 | 1.03 | 1.46 | 1.63 |

| **Image_Name** | **Method** | **N** | **Mean** | **Std Dev** | **Std Err** | **Minimum** | **Maximum** |
| --- | --- | --- | --- | --- | --- | --- | --- |
| **G0/G1** |  | 6 | 2.1758 | 0.2172 | 0.0887 | 1.8230 | 2.4513 |
| **S** |  | 6 | 1.2750 | 0.2499 | 0.1020 | 1.0192 | 1.6304 |
| **Diff (1-2)** | **Pooled** |  | 0.9008 | 0.2341 | 0.1352 |  |  |
| **Diff (1-2)** | **Satterthwaite** |  | 0.9008 |  | 0.1352 |  |  |

| **Image_Name** | **Method** | **Mean** | **95% CL Mean** | | **Std Dev** | **95% CL Std Dev** | |
| --- | --- | --- | --- | --- | --- | --- | --- |
| **G0/G1** |  | 2.1758 | 1.9478 | 2.4038 | 0.2172 | 0.1356 | 0.5328 |
| **S** |  | 1.2750 | 1.0128 | 1.5373 | 0.2499 | 0.1560 | 0.6128 |
| **Diff (1-2)** | **Pooled** | 0.9008 | 0.5996 | 1.2019 | 0.2341 | 0.1636 | 0.4109 |
| **Diff (1-2)** | **Satterthwaite** | 0.9008 | 0.5988 | 1.2027 |  |  |  |

| **Method** | **Variances** | **DF** | **t Value** | **Pr > \|t\|** |
| --- | --- | --- | --- | --- |
| **Pooled** | Equal | 10 | 6.66 | <.0001 |
| **Satterthwaite** | Unequal | 9.8103 | 6.66 | <.0001 |

| **Equality of Variances** | | | | |
| --- | --- | --- | --- | --- |
| **Method** | **Num DF** | **Den DF** | **F Value** | **Pr > F** |
| **Folded F** | 5 | 5 | 1.32 | 0.7662 |

# Figure 4C Western quant

|  | | **N** | **NMiss** | **Min** | **Mean** | **Std** | **Median** | **Q1** | **Q3** | **Max** |
| --- | --- | --- | --- | --- | --- | --- | --- | --- | --- | --- |
| **CEP164** | **CEP164 siRNA** | 3 | 0 | 0.70 | 0.72 | 0.03 | 0.71 | 0.70 | 0.76 | 0.76 |
|  | **Control siRNA** | 3 | 0 | 1.01 | 1.07 | 0.05 | 1.09 | 1.01 | 1.12 | 1.12 |

P=0.0006

|  | | **N** | **NMiss** | **Min** | **Mean** | **Std** | **Median** | **Q1** | **Q3** | **Max** |
| --- | --- | --- | --- | --- | --- | --- | --- | --- | --- | --- |
| **CENTRIN2** | **CEP164 siRNA** | 3 | 0 | 0.66 | 0.68 | 0.02 | 0.69 | 0.66 | 0.70 | 0.70 |
|  | **Control siRNA** | 3 | 0 | 1.08 | 1.14 | 0.09 | 1.09 | 1.08 | 1.23 | 1.23 |

P=0.0009

|  | | **N** | **NMiss** | **Min** | **Mean** | **Std** | **Median** | **Q1** | **Q3** | **Max** |
| --- | --- | --- | --- | --- | --- | --- | --- | --- | --- | --- |
| **ARL13B** | **CEP164 siRNA** | 3 | 0 | 0.67 | 0.69 | 0.02 | 0.68 | 0.67 | 0.71 | 0.71 |
|  | **Control siRNA** | 3 | 0 | 0.86 | 0.90 | 0.05 | 0.88 | 0.86 | 0.95 | 0.95 |

P=0.0025

# Figure S2

Negative binomial model was performed to compare the two groups.

## Arl13B_in_sub_G0

|  | | **N** | **NMiss** | **Min** | **Mean** | **Std** | **Median** | **Q1** | **Q3** | **Max** |
| --- | --- | --- | --- | --- | --- | --- | --- | --- | --- | --- |
| **Arl13B_in_sub_G0** | **Control** | 4 | 0 | 558.00 | 939.00 | 363.42 | 896.00 | 666.50 | 1211.50 | 1406.00 |
|  | **G0G1 inh** | 4 | 0 | 1511.00 | 2019.75 | 445.07 | 2074.00 | 1647.00 | 2392.50 | 2420.00 |

| **Group Least Squares Means** | | | | | | | |
| --- | --- | --- | --- | --- | --- | --- | --- |
| **Group** | **Estimate** | **Standard Error** | **z Value** | **Pr > \|z\|** | **Alpha** | **Lower** | **Upper** |
| **Control** | 6.8448 | 0.1372 | 49.88 | <.0001 | 0.05 | 6.5758 | 7.1138 |
| **G0G1 inh** | 7.6107 | 0.1367 | 55.67 | <.0001 | 0.05 | 7.3428 | 7.8787 |

| **Differences of Group Least Squares Means** | | | | | | | | |
| --- | --- | --- | --- | --- | --- | --- | --- | --- |
| **Group** | **_Group** | **Estimate** | **Standard Error** | **z Value** | **Pr > \|z\|** | **Alpha** | **Lower** | **Upper** |
| **Control** | **G0G1 inh** | -0.7659 | 0.1937 | -3.95 | <.0001 | 0.05 | -1.1456 | -0.3863 |

Estimated mean and 95% CI

| Group | Mean and 95% CI |
| --- | --- |
| Control | 939 (718, 1229) |
| G0G1 inh | 2020 (1545, 2640) |

## Arl13B_in_G0

|  | | **N** | **NMiss** | **Min** | **Mean** | **Std** | **Median** | **Q1** | **Q3** | **Max** |
| --- | --- | --- | --- | --- | --- | --- | --- | --- | --- | --- |
| **Arl13B_in_G0** | **Control** | 4 | 0 | 4513.00 | 11667.00 | 8264.76 | 11437.50 | 4517.00 | 18817.00 | 19280.00 |
|  | **G0G1 inh** | 4 | 0 | 25440.00 | 25629.25 | 213.21 | 25587.00 | 25461.00 | 25797.50 | 25903.00 |

| **Group Least Squares Means** | | | | | | | |
| --- | --- | --- | --- | --- | --- | --- | --- |
| **Group** | **Estimate** | **Standard Error** | **z Value** | **Pr > \|z\|** | **Alpha** | **Lower** | **Upper** |
| **Control** | 9.3645 | 0.2382 | 39.31 | <.0001 | 0.05 | 8.8976 | 9.8315 |
| **G0G1 inh** | 10.1515 | 0.2382 | 42.61 | <.0001 | 0.05 | 9.6846 | 10.6184 |

| **Differences of Group Least Squares Means** | | | | | | | | |
| --- | --- | --- | --- | --- | --- | --- | --- | --- |
| **Group** | **_Group** | **Estimate** | **Standard Error** | **z Value** | **Pr > \|z\|** | **Alpha** | **Lower** | **Upper** |
| **Control** | **G0G1 inh** | -0.7870 | 0.3369 | -2.34 | 0.0195 | 0.05 | -1.4473 | -0.1266 |

Estimated mean and 95% CI

| Group | Mean and 95% CI |
| --- | --- |
| Control | 11667 (7314, 18610) |
| G0G1 inh | 25630 (16068, 40880) |

## Arl13B_in_G1

|  | | **N** | **NMiss** | **Min** | **Mean** | **Std** | **Median** | **Q1** | **Q3** | **Max** |
| --- | --- | --- | --- | --- | --- | --- | --- | --- | --- | --- |
| **Arl13B_in_G1** | **Control** | 4 | 0 | 3799.00 | 10216.50 | 7377.55 | 10203.50 | 3827.50 | 16605.50 | 16660.00 |
|  | **G0G1 inh** | 4 | 0 | 22384.00 | 22654.00 | 344.19 | 22550.50 | 22402.50 | 22905.50 | 23131.00 |

| **Group Least Squares Means** | | | | | | | |
| --- | --- | --- | --- | --- | --- | --- | --- |
| **Group** | **Estimate** | **Standard Error** | **z Value** | **Pr > \|z\|** | **Alpha** | **Lower** | **Upper** |
| **Control** | 9.2318 | 0.2443 | 37.80 | <.0001 | 0.05 | 8.7530 | 9.7105 |
| **G0G1 inh** | 10.0281 | 0.2442 | 41.06 | <.0001 | 0.05 | 9.5494 | 10.5068 |

| **Differences of Group Least Squares Means** | | | | | | | | |
| --- | --- | --- | --- | --- | --- | --- | --- | --- |
| **Group** | **_Group** | **Estimate** | **Standard Error** | **z Value** | **Pr > \|z\|** | **Alpha** | **Lower** | **Upper** |
| **Control** | **G0G1 inh** | -0.7963 | 0.3454 | -2.31 | 0.0211 | 0.05 | -1.4733 | -0.1193 |

Estimated mean and 95% CI

| Group | Mean and 95% CI |
| --- | --- |
| Control | 11667 (7314, 18610) |
| G0G1 inh | 25630 (16068, 40880) |

## Arl13B_in_S

|  | | **N** | **NMiss** | **Min** | **Mean** | **Std** | **Median** | **Q1** | **Q3** | **Max** |
| --- | --- | --- | --- | --- | --- | --- | --- | --- | --- | --- |
| **Arl13B_in_S** | **Control** | 4 | 0 | 5424.00 | 14900.75 | 10924.77 | 14760.00 | 5442.00 | 24359.50 | 24659.00 |
|  | **G0G1 inh** | 4 | 0 | 33185.00 | 33934.00 | 701.94 | 33931.50 | 33349.50 | 34518.50 | 34688.00 |

| **Group Least Squares Means** | | | | | | | |
| --- | --- | --- | --- | --- | --- | --- | --- |
| **Group** | **Estimate** | **Standard Error** | **z Value** | **Pr > \|z\|** | **Alpha** | **Lower** | **Upper** |
| **Control** | 9.6092 | 0.2489 | 38.60 | <.0001 | 0.05 | 9.1213 | 10.0970 |
| **G0G1 inh** | 10.4322 | 0.2489 | 41.91 | <.0001 | 0.05 | 9.9443 | 10.9200 |

| **Differences of Group Least Squares Means** | | | | | | | | |
| --- | --- | --- | --- | --- | --- | --- | --- | --- |
| **Group** | **_Group** | **Estimate** | **Standard Error** | **z Value** | **Pr > \|z\|** | **Alpha** | **Lower** | **Upper** |
| **Control** | **G0G1 inh** | -0.8230 | 0.3520 | -2.34 | 0.0194 | 0.05 | -1.5130 | -0.1331 |

Estimated mean and 95% CI

| Group | Mean and 95% CI |
| --- | --- |
| Control | 14901 (9148, 24270) |
| G0G1 inh | 33934 (20833, 55271) |

## Arl13B_in_G2M

|  | | **N** | **NMiss** | **Min** | **Mean** | **Std** | **Median** | **Q1** | **Q3** | **Max** |
| --- | --- | --- | --- | --- | --- | --- | --- | --- | --- | --- |
| **Arl13B_in_G2M** | **Control** | 4 | 0 | 6615.00 | 17534.50 | 12286.72 | 17114.00 | 6927.50 | 28141.50 | 29295.00 |
|  | **G0G1 inh** | 4 | 0 | 38026.00 | 39317.75 | 1561.58 | 38882.00 | 38183.00 | 40452.50 | 41481.00 |

| **Group Least Squares Means** | | | | | | | |
| --- | --- | --- | --- | --- | --- | --- | --- |
| **Group** | **Estimate** | **Standard Error** | **z Value** | **Pr > \|z\|** | **Alpha** | **Lower** | **Upper** |
| **Control** | 9.7719 | 0.2351 | 41.56 | <.0001 | 0.05 | 9.3111 | 10.2328 |
| **G0G1 inh** | 10.5794 | 0.2351 | 45.00 | <.0001 | 0.05 | 10.1186 | 11.0403 |

| **Differences of Group Least Squares Means** | | | | | | | | |
| --- | --- | --- | --- | --- | --- | --- | --- | --- |
| **Group** | **_Group** | **Estimate** | **Standard Error** | **z Value** | **Pr > \|z\|** | **Alpha** | **Lower** | **Upper** |
| **Control** | **G0G1 inh** | -0.8075 | 0.3325 | -2.43 | 0.0152 | 0.05 | -1.4592 | -0.1558 |

Estimated mean and 95% CI

| Group | Mean and 95% CI |
| --- | --- |
| Control | 17534 (11060, 27800) |
| G0G1 inh | 39316 (24800, 62336) |
