# Supplementary figures and images for: Brain microvascular endothelial cells possess a second cilium that arises from the daughter centriole
Source: Front Mol Biosci. 2023 Nov 6;10:1250016. doi: 10.3389/fmolb.2023.1250016 (PMC10657992; doi:10.3389/fmolb.2023.1250016)

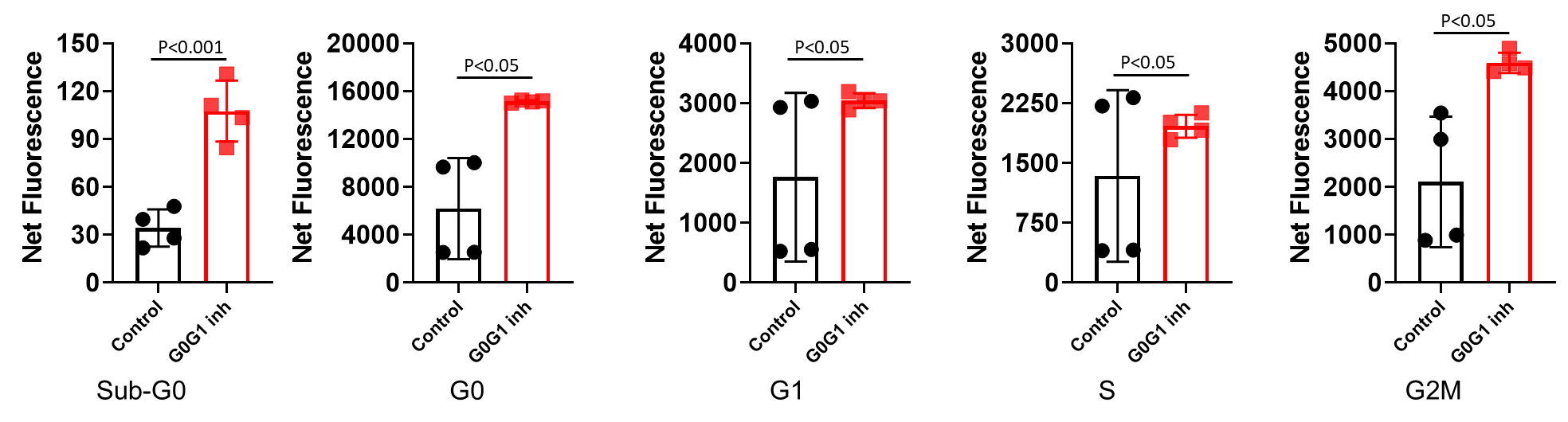

Supplement: Supplementary file 2 [file Image3.TIF]

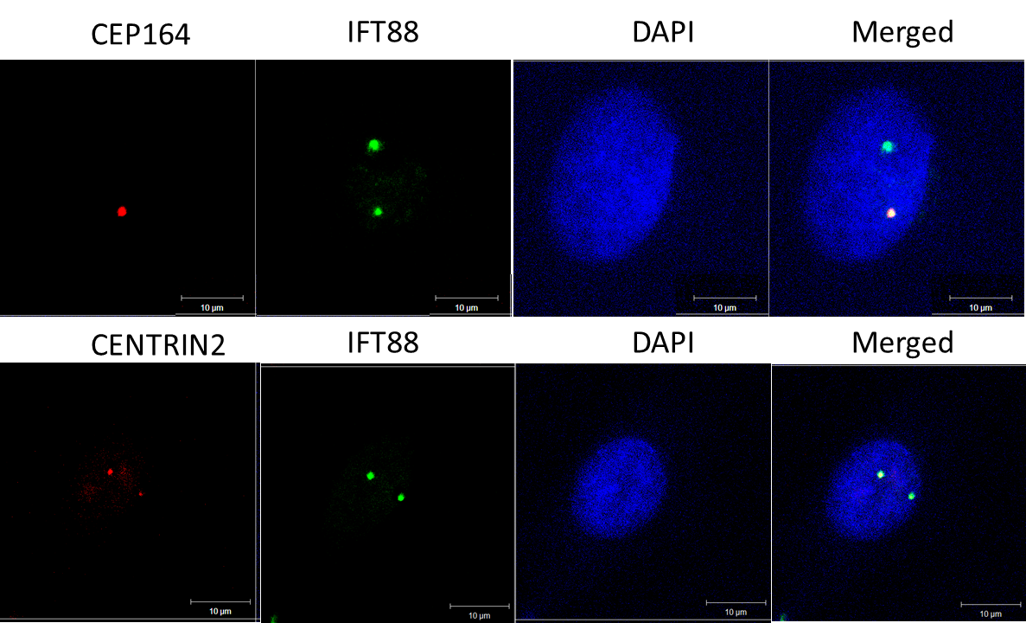

Supplement: Supplementary file 3 [file Image2.TIF]

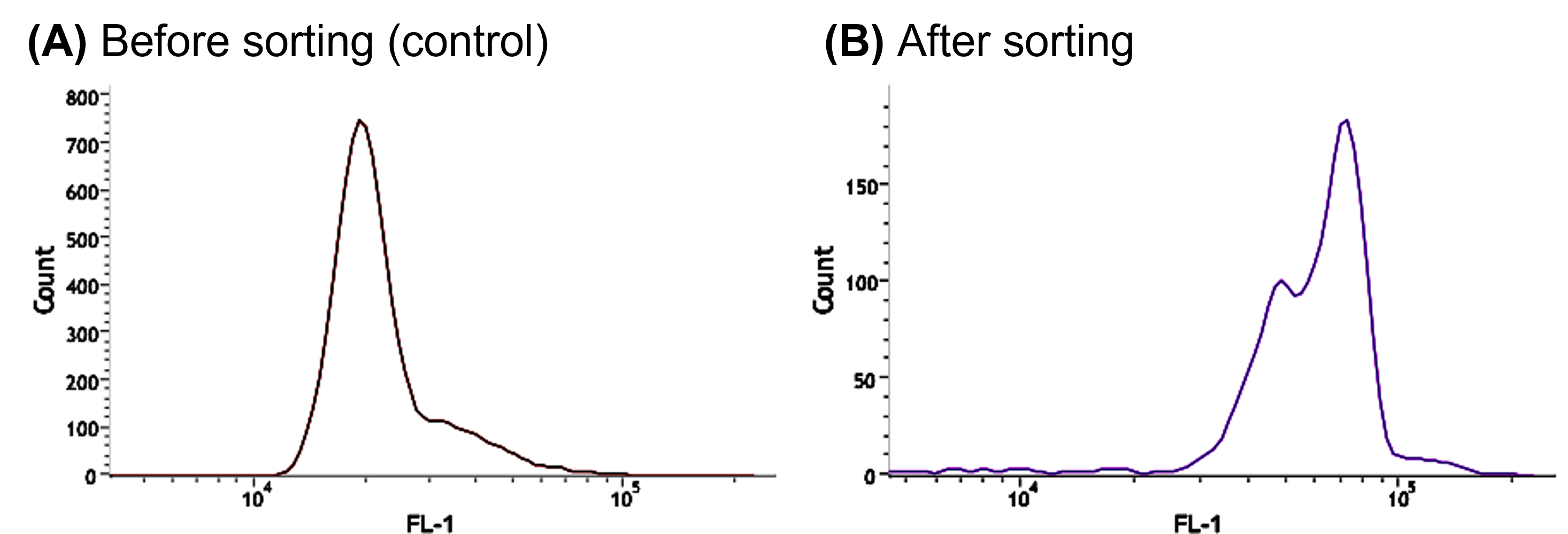

Supplement: Supplementary file 4 [file Image1.TIF]
